# Lead Dynamics in Saline Aquaponics: Tissue Accumulation in *Litopenaeus vannamei* and *Sesuvium portulacastrum* and Root-Associated Bacterial Communities

**DOI:** 10.3390/ani16193009

**Published:** 2026-09-24

**Authors:** Mariel Gullian Klanian, María José Sánchez Solís, Joel Cutz de Ocampo

**Affiliations:** Experimental Unit, Universidad Marista de Mérida, Periférico Norte Tablaje Catastral 13941, Temozón Nte, Mérida 97300, Yucatán, Mexico; msanchez@marista.edu.mx (M.J.S.S.); jcutz@marista.edu.mx (J.C.d.O.)

**Keywords:** lead dynamics, saline aquaponics, *Sesuvium portulacastrum*, *Litopenaeus vannamei*, physiological biomarkers, root-associated bacterial communities

## Abstract

Lead (Pb) contamination in coastal groundwater poses a potential concern for aquaculture systems relying on saline water sources; however, biological patterns associated with naturally Pb-enriched water in integrated saline aquaponic systems remain poorly characterized. This exploratory study described Pb dynamics, plant tissue accumulation, shrimp physiological biomarkers, and root-associated microbiota in a single saline aquaponic system supplied with naturally Pb-enriched seawater (3.01 ± 1.03 µg/L) over eight weeks. Dissolved Pb concentrations were 75 ± 20% lower at the hydroponic outlet than at the sampled inlet, indicating system-level Pb retention. Pb accumulation in *Sesuvium portulacastrum* tissues indicates plant participation in Pb retention; however, the specific contribution of the plant could not be separated from retention by sedimentation, biofilter media, surfaces, or biofilms. *Litopenaeus vannamei* concentrated Pb predominantly in the cephalothorax and exhibited temporal fluctuations in hemocyte counts, osmolality, lysozyme activity, and catalase activity. Root-associated bacterial communities of aquaponic plants showed slightly higher genus richness (116 genera, H′ = 4.24) than dune reference plants (106 genera, H′ = 4.00), together with compositional differences involving saline- and metal-associated taxa. Because the study was based on a single aquaponic system without an uncontaminated parallel system or independent system-level replication, the observed patterns describe within-system temporal variation and cannot be attributed specifically to Pb. Nevertheless, the study illustrates the value of integrating plant, shrimp, and microbial indicators to characterize metal dynamics in saline aquaponic systems and provides exploratory evidence to guide future replicated studies of phytotechnology applications in contaminated coastal settings.

## Simple Summary

Coastal communities in tropical regions may rely on groundwater containing trace concentrations of heavy metals, including lead (Pb). When this water is used for aquaculture, Pb can enter the production system and accumulate in farmed organisms. This study monitored an eight-week saline aquaponic system supplied with naturally Pb-enriched water and examined Pb concentrations in water, the salt-tolerant plant *Sesuvium portulacastrum*, and farmed white shrimp (*Litopenaeus vannamei*), together with root-associated bacterial communities. Dissolved Pb concentrations were, on average, 75% lower at the hydroponic outlet than at the sampled inlet. This difference represents retention by the aquaponic system as a whole and may involve plant uptake, sedimentation, biofilter media, pipe surfaces, and biofilms. Pb was detected in plant tissues, showing that *S. portulacastrum* participated in Pb retention, although its specific contribution could not be quantified. Shrimp accumulated Pb mainly in the cephalothorax and showed temporal variation in several physiological indicators. Root-associated bacterial communities also differed descriptively between aquaponic plants and dune reference plants. Because the study involved one aquaponic system without an uncontaminated control system, these observations cannot establish Pb-specific effects. Nevertheless, the combined assessment of water, plants, shrimp, and microbial communities provides an exploratory framework for monitoring metal dynamics in saline aquaponics.

## 1. Introduction

Heavy metal contamination in aquatic environments, particularly by lead (Pb), poses a significant threat to public health, food safety, and the sustainability of productive systems [1]. The Pacific white shrimp *Litopenaeus vannamei*, a globally important aquaculture species, is susceptible to Pb bioaccumulation, which compromises its suitability for human consumption. Although Pb concentrations reported in farmed shrimp generally remain below FAO/WHO limits [2], the World Health Organization emphasizes that no level of Pb exposure is safe, given its capacity to accumulate in bone tissue with a half-life of decades [3]. This underscores the need for continuous monitoring and mitigation strategies that minimize Pb transfer to aquaculture products.

In intensive recirculating systems such as aquaponics, contaminants entering through the water source can circulate within the closed loop, accumulating in both plants and animals and increasing toxicological risks. In regions supplied by untreated coastal groundwater with naturally elevated Pb, these risks become particularly relevant. However, biological patterns occurring in plants, animals, and associated microbiota under chronic, low-level, field-relevant Pb-enriched conditions remain poorly characterized, and practical guidance for monitoring Pb stress in metal-impacted aquaponic systems is limited.

Halophytic plants such as *S. portulacastrum* have gained attention as candidates for phytoremediation in saline environments because they tolerate high salinity and chemical stress, produce osmoprotectants, and can detoxify metals through vacuolar sequestration, metal-binding proteins, and antioxidant metabolism [4,5]. Beyond their intrinsic traits, their rhizosphere microbiome can contribute to heavy metal immobilization through biosorption, enzyme-mediated precipitation, phosphate solubilization, or pH modification [6]. Recent studies suggest that manipulating these microbial communities, via natural selection or engineered consortia, can enhance phytoremediation efficiency [7], but the functional and structural dynamics of this plant–microbe partnership under saline aquaponic conditions are still largely unknown.

From an animal health perspective, *L. vannamei* represents a sensitive bioindicator due to its rapid metabolism and well-characterized immune system. Experimental exposures have shown that Pb concentrations as low as 0.1 mg/L reduce hemocyte counts and impair respiratory burst activity [8], while other studies report osmoregulatory disruption, elevated hepatic biomarkers, and metabolic stress [9,10]. These responses reflect systemic impacts that compromise physiological stability and immunocompetence and may be exacerbated in recirculating systems where exposure is chronic rather than episodic.

Despite the growing adoption of aquaponics as a sustainable food production strategy, integrative assessments that simultaneously evaluate water chemistry, plant tissue Pb accumulation, shrimp physiological biomarkers, and root-associated bacterial communities in aquaponic systems supplied with naturally Pb-enriched water are virtually absent. Addressing this gap may support the development of monitoring strategies for aquaculture systems operating with metal-affected water sources.

This exploratory study characterizes Pb dynamics and associated biological patterns in a saline aquaponic system supplied with naturally Pb-enriched seawater. Specifically, we (i) quantify inlet–outlet differences in dissolved Pb concentrations across the hydroponic unit and document Pb accumulation in *S. portulacastrum* tissues; (ii) describe tissue Pb concentrations and temporal variation in physiological and immune biomarkers of *L. vannamei*; and (iii) characterize root-associated bacterial communities at the end of the monitoring period. By integrating plant, animal, and microbial endpoints, this work provides preliminary evidence for multiscale monitoring of metal dynamics in saline aquaponic systems and identifies priorities for future replicated studies.

## 2. Materials and Methods

### 2.1. Study Design and Water Sources

This exploratory study characterized Pb dynamics and associated biological patterns in a saline aquaponic system supplied with naturally Pb-enriched water. The aquaponic system was located in Mérida, Yucatán, Mexico (21°05′31″ N, 89°62′38″ W), and supplied with seawater pumped from a coastal well at 95 m depth. This source, characterized by a salinity of 29 PSU and a mean Pb concentration of 3.01 ± 1.03 µg/L, provided naturally elevated levels without artificial dosing, thereby ensuring environmentally relevant exposure conditions.

The aquaponic system followed a nutrient film technique (NFT) design, consisting of one aquaculture tank (1.05 m^3^) connected to four PVC hydroponic pipes with plant baskets. Water flowed through a sedimentation chamber, a biofilter with K1 media, and then into the hydroponic channels before returning to the tank, forming a closed recirculation loop. Flow was maintained at 1–3 L/min to create a thin water film that optimized root oxygenation and nutrient uptake.

A total of 66 juvenile *L. vannamei* (35 ± 5.9 g) were stocked in the aquaculture tank at 2.2 kg/m^3^ and fed twice daily with a commercial diet (30% protein, 6–8% lipids), adjusting rations to body weight. Twenty-four *S. portulacastrum* cuttings from a coastal dune were acclimated for three weeks and placed individually in hydroponic baskets. Plants were supported with sponges, and overhead grids prevented creeping stems from obstructing water flow. The 24 *S. portulacastrum* plants remained in the aquaponic system throughout the eight-week monitoring period. Weekly plant sampling was non-lethal: leaf, stem, and root portions were collected from selected living plants without removing whole individuals from the system.

The system was monitored for eight weeks, with weekly sampling of water, *S. portulacastrum*, and *L. vannamei* to characterize Pb dynamics and associated physiological patterns. At each weekly sampling point, hemolymph was collected from five individual shrimp, after which the same shrimp were euthanized and used for Pb determination in tail muscle and cephalothorax. Thus, a total of 40 shrimp were sampled during weeks 1–8 from the initial stock of 66 individuals. All procedures followed internationally recognized principles for the ethical use of aquatic invertebrates in research. Shrimp were handled with minimal stress and euthanized by rapid chilling on ice, a method considered humane for crustaceans [11].

Due to the environmental nature of this study, the seawater used in the aquaponic system contained naturally elevated Pb levels, making it impossible to establish a true uncontaminated control tank under identical operational conditions. Therefore, baseline plant and shrimp values obtained prior to exposure or from natural dune ecosystems were used solely as ecological reference points and not for inferential statistics. All analyses were thus focused on temporal patterns within the Pb-enriched system and on exploratory associations among water chemistry, bioaccumulation, physiological biomarkers, and microbiome structure.

The experimental unit was the single saline aquaponic system. Individual shrimp and plant samples collected at each weekly sampling point were within-system biological subsamples used to characterize organism-level and temporal variation. They were not independent system-level replicates of Pb exposure. Accordingly, this study was designed as an exploratory observational assessment, and the statistical analyses were interpreted as describing temporal patterns within the monitored system rather than as confirmatory tests of Pb-specific effects.

### 2.2. Water Quality Monitoring and Lead Analysis

Water samples were collected weekly for eight weeks at the biofilter inlet and hydroponic outlet. Nutrients, including ammonium nitrogen (NH_3_-N), nitrite nitrogen (NO_2_-N), nitrate nitrogen (NO_3_-N), total phosphorus (TP), and potassium (K^+^), were quantified spectrophotometrically using a Hach DR2800 instrument (Loveland, CO, USA) following standard Hach methods [12].

Lead (Pb) concentrations were determined using the 5-Carboxy-PADAP colorimetric procedure, with absorbance measured at 548 nm [13]. This method was selected because atomic spectrometric instrumentation, including AAS, ICP-OES, and ICP-MS, was not available at the institution at the time of analysis. To reduce potential interference from co-occurring metal ions, including Cu^2+^, Zn^2+^, and Fe^3+^, EDTA was added as a masking agent during the colorimetric reaction. Calibration curves were prepared using Pb standards in ultrapure water and in digested tissue matrix to evaluate potential matrix-related differences in response.

The method was used as a screening approach and was not validated against certified reference materials or independently confirmed by atomic spectrometric analysis. Therefore, measured Pb concentrations should be interpreted with caution, particularly when used for regulatory food-safety comparisons. All measurements were performed in analytical triplicate, and results were expressed as µg/L for nutrients and Pb.

### 2.3. Lead Quantification in Plant and Shrimp Tissues

Lead accumulation was assessed weekly in *S. portulacastrum* (leaf, stem, and root) and *L. vannamei* (cephalothorax and tail muscle). For plants, leaf, stem, and root portions were sampled non-lethally each week from five living individuals selected from the 24 plants maintained in the aquaponic system. Whole plants were not removed, and individual plants could be sampled again in subsequent weeks after regeneration. For shrimp, five individual animals were sampled each week as within-system biological subsamples. Hemolymph was collected first for physiological and immune biomarker analyses, after which the same individuals were euthanized and dissected for Pb determination in the cephalothorax and tail muscle. Thus, hemolymph biomarker measurements and tissue Pb concentrations were paired within each shrimp at each weekly sampling time point.

For each biological sample, Pb quantification was performed in analytical triplicate to assess measurement precision; the mean of the three readings was used as the value for that individual. The *n* = 5 reported in tables refers to five individual plant subsamples per tissue per week and five individuqqqqqal shrimp subsamples *per* tissue per week, all collected within the single aquaponic system. These values represent within-system biological variation and were not treated as independent system-level replicates of Pb exposure. Analytical triplicates were averaged within each individual and were not treated as independent observations. Plant and shrimp tissues (~1 g) were digested using a nitric acid–hydrogen peroxide protocol, and Pb was quantified spectrophotometrically with the 5-Carboxy-PADAP method (λ = 548 nm).

Baseline plant values correspond to *S. portulacastrum* collected from a natural coastal dune ecosystem with no known Pb contamination, while shrimp baselines were obtained one week before their introduction into the Pb-enriched aquaponic system. These measurements serve only as environmental and physiological reference points.

### 2.4. Physiological and Immune Biomarkers in Litopenaeus vannamei

To monitor physiological and immune biomarkers, hemolymph was collected weekly from five individual shrimp per time point. Each shrimp was sampled once only and represented one within-system biological subsample. Immediately after hemolymph collection, the same individuals were euthanized and dissected for Pb determination in tail muscle and cephalothorax, as described in Section 2.3. Thus, hemolymph biomarker measurements and tissue Pb concentrations were paired within each shrimp at each weekly sampling point.

Each hemolymph sample was analyzed in analytical triplicate to assess measurement precision, and the mean of the three readings was used as the value for the corresponding individual shrimp. Additionally, hemolymph was collected from five shrimp one week before transfer to the Pb-enriched aquaponic system to obtain pre-exposure physiological and immune reference values for contextual comparison.

Osmolarity was measured with a vapor pressure osmometer. Total hemocyte count (THC) was obtained with a Neubauer chamber. Oxidative and immune biomarkers were determined spectrophotometrically: reactive oxygen species (ROS) by NBT reduction [14], catalase (CAT) activity by H_2_O_2_ decomposition at 240 nm [15], and lysozyme (LZM) activity turbidimetrically using *Micrococcus lysodeikticus*.

### 2.5. Antioxidant Analysis in Sesuvium portulacastrum

Antioxidant responses in *S. portulacastrum* were evaluated weekly in leaf tissue. Each week, leaf portions were collected non-lethally from five living plants selected from the 24 individuals maintained in the aquaponic system. Although leaf, stem, and root tissues were sampled separately for Pb determination, antioxidant assays were conducted exclusively in leaves. Leaf samples were individually ground in liquid nitrogen, and approximately 1 g of tissue from each plant was used for enzymatic and non-enzymatic analyses. Whole plants were not removed, and some individuals could contribute leaf samples at more than one weekly time point after regeneration. Therefore, the five values reported per week represent within-system leaf subsamples collected at that sampling time point; temporal analyses were interpreted as exploratory because repeated sampling of individual plants across weeks could not be fully excluded. These samples describe individual-level variation within the monitored system and do not represent independent system-level replicates of Pb exposure. All assays were performed in analytical triplicate to assess measurement precision, and the mean of the three readings was used as the value for each plant. Enzymatic antioxidants were assayed spectrophotometrically: peroxidase (POD) at 470 nm [15], catalase (CAT) at 240 nm [16], and ascorbate peroxidase (APX) at 290 nm [17]. Non-enzymatic antioxidants were quantified as follows: lycopene (LYP) by hexane–acetone extraction at 502 nm [18] and ascorbic acid (AA) by the DTT/ascorbate oxidase method at 265 nm [19]. Protein concentration was determined by the Lowry method [20]. Baseline antioxidant levels were obtained from *S. portulacastrum* collected at a natural coastal dune site and processed using the same analytical procedures.

### 2.6. Root Microbiome Analysis of Sesuvium portulacastrum via 16S rRNA Sequencing

Root-associated bacterial communities of *S. portulacastrum* were characterized only at week 8. Two groups were included: plants from the Pb-enriched aquaponic system (aquaponic group, *n* = 3) and plants collected from a coastal dune reference site representing the species’ natural environmental context (dune reference group, *n* = 3). Each group included three biological replicates, with each replicate corresponding to one individual plant. This analysis represents a single end-point descriptive comparison between plants grown in the aquaponic system and plants collected from the dune reference site; it was not designed to isolate the effects of Pb from other environmental differences between the two sites.

Root samples were rinsed with sterile PBS, frozen in liquid nitrogen, and ground to a fine powder. DNA was extracted with the Quick-DNA™ Soil Microbe Miniprep Kit (Zymo Research, Irvine, CA, USA) following the manufacturer’s protocol. DNA quality was checked by spectrophotometry and agarose gel electrophoresis. The V3–V4 region of the 16S rRNA gene was amplified with primers 341F/805R; libraries were prepared with the Quick-16S™ NGS Library Prep Kit (Zymo Research) and sequenced on an Illumina MiSeq™ platform (2 × 300 bp).

### 2.7. Microbiome Data Analysis

Raw reads were processed using the DADA2 pipeline (v1.18) for quality control and amplicon sequence variant (ASV) inference, and taxonomic assignment was performed against the SILVA 138 database. Root samples from dune reference plants were included for descriptive environmental comparison only and were not treated as an experimental control group. Taxa with relative abundance >1% in at least one sample were retained for visualization of community composition. Genus-level relative abundances were calculated from QIIME2 output and compared descriptively between the aquaponic and dune reference groups. Shannon diversity indices (H′) were calculated separately for individual samples at the ASV level and summarized by group; genus-level diversity values were also calculated descriptively for the group comparison. No formal between-group hypothesis testing was performed because the comparison was limited to one end-point sampling event and the groups differed in environmental context.

### 2.8. Statistical Analysis

Descriptive statistics were used to summarize all variables and are reported as mean ± SE. The experimental unit was the single saline aquaponic system. Individual shrimp and plant samples collected within each week were within-system biological subsamples and were used to describe organism-level variation; they were not independent system-level replicates of Pb exposure. Because the study included one aquaponic system without an uncontaminated system-level control or independent system replication, all statistical analyses were exploratory and focused on temporal variation within the monitored system. Accordingly, *p*-values are reported only as descriptive indicators of within-system temporal heterogeneity and do not support causal or system-level inferential conclusions regarding Pb exposure.

Exploratory comparisons of tissue- and week-associated variation in plant and shrimp Pb concentrations were performed using the Aligned Rank Transform (ART) for factorial analysis of ranked observations [21]. For time-series variables measured within the aquaponic system, including plant antioxidant variables and shrimp physiological biomarkers, temporal differences among weeks were evaluated using Kruskal–Wallis tests. For plant variables, results were interpreted cautiously because plant tissue sampling was non-lethal and some individuals may have contributed samples at more than one weekly time point. Spearman correlations were used to explore associations between weekly mean water-quality variables, Pb retention, tissue Pb concentrations, and physiological or antioxidant variables, including same-week and one-week lagged comparisons. Weekly mean values were used because water-quality measurements were obtained at the system level. Raw *p*-values were adjusted using the Benjamini–Hochberg false discovery rate (FDR) method, and adjusted values are reported as q-values. Analyses were conducted using XLSTAT (Addinsoft, Paris, France) and Python (v3.11).

## 3. Results

### 3.1. Interactions Between Water Nutrients and Lead Dynamics in the Aquaponic System

Nutrient concentrations fluctuated throughout the experiment (Table S1). Nitrate was consistently higher at the outlet than at the inlet (e.g., 5.60 vs. 3.00 mg/L in week 1), while ammonium (NH_3_) was consistently lower at the outlet (e.g., 0.360 vs. 0.270 mg/L in week 2). Occasional nitrite peaks were observed (e.g., week 2: 0.326 vs. 0.245 mg/L). Total phosphorus (TP) ranged from 0.69 to 3.27 mg/L. Concentrations of potassium were substantially higher than those of other nutrients (72.5–370.7 mg/L), with large reductions between inlet and outlet (e.g., 370.7 to 90.0 mg/L in week 2).

Weekly measurements showed lower dissolved Pb concentrations at the hydroponic outlet than at the sampled biofilter inlet (Table 1). Inlet Pb concentrations ranged from 2.50 ± 0.14 to 13.00 ± 0.75 µg/L (mean 6.94 ± 3.4 µg/L), whereas outlet Pb concentrations ranged from 0.00 to 4.00 ± 0.23 µg/L (mean 1.44 ± 1.3 µg/L). The mean inlet–outlet difference corresponded to 75 ± 20% system-level Pb retention. The maximum weekly difference was 100% in week 6, whereas the minimum was 36.4% in week 3. Outlet Pb concentrations were lower than inlet concentrations at all sampling points.

The observed inlet–outlet differences represent system-level Pb retention and may reflect the combined contributions of *S. portulacastrum*, sedimentation, biofilter media, pipe surfaces, and biofilms. Pb accumulation in plant tissues supports the participation of *S. portulacastrum* in Pb retention; however, because no plant-free control system or complete Pb mass balance was available, the specific contribution of the plant could not be quantified.

Pb retention correlated negatively with inlet ammonium (R = −0.55) and total phosphorus (R = −0.49), and positively with outlet ammonium (R = 0.42). An exploratory multivariable model accounted for 55.5% of the observed variation in system-level Pb retention; given the limited number of weekly observations, the model was not used for predictive inference.

### 3.2. Tissue Pb Concentrations and Antioxidant Variables in Sesuvium portulacastrum

During the eight-week monitoring period, Pb was detected in leaves, stems, and roots of *S. portulacastrum* plants maintained in the Pb-enriched aquaponic system (Table 2). Leaf concentrations ranged from 37.77 ± 2.2 µg/100 g in week 5 to 197.88 ± 11.4 µg/100 g in week 8. Stem concentrations ranged from 53.59 ± 3.1 µg/100 g in week 6 to 243.34 ± 14.0 µg/100 g in week 5. Root concentrations ranged from 65.13 ± 3.8 µg/100 g in week 4 to 207.30 ± 4.2 µg/100 g in week 5. ART ANOVA (*n* = 5 samples per tissue–week) detected a significant effect of week (F = 4.63, *p* = 0.00031) and a significant week × tissue interaction (F = 24.08, *p* < 0.001). Tissue did not differ significantly overall (F = 0.45, *p* = 0.639). Because some living plants may have contributed samples at more than one time point, these temporal results are interpreted as exploratory.

Antioxidant variables measured in leaf tissue showed temporal variation during the eight-week monitoring period (Table 3). POD (H = 28.31, *p* = 0.0002), APX (H = 27.50, *p* = 0.0003), CAT (H = 26.16, *p* = 0.0005), and AA (H = 20.88, *p* = 0.0040) differed among weeks, whereas LYP did not (H = 13.49, *p* = 0.0610). POD showed its highest mean value in week 7 (93.66 ± 5.51 U/mg), APX in week 2 (30.95 ± 1.75 U/mg), CAT in week 8 (313.51 ± 16.58 U/mg), and AA in week 5 (5.14 ± 1.17 µg/mg). LYP showed no statistically significant temporal variation. Because plant tissue sampling was non-lethal and some plants may have contributed samples at more than one weekly time point, these temporal patterns are interpreted as exploratory.

Exploratory Spearman correlation analysis identified a positive raw association between leaf CAT activity and leaf Pb concentration (*ρ* = 0.74, *p* = 0.036; *n* = 8 weekly means). After Benjamini–Hochberg adjustment across four tests, this association did not remain statistically significant (*q* = 0.146). No other enzyme–tissue or enzyme–water associations remained significant after multiple-testing correction.

### 3.3. Tissue Pb Concentrations and Physiological Biomarkers in Litopenaeus vannamei

Pb was quantified in tail muscle and cephalothorax of *L. vannamei* over the eight-week monitoring period (Table 4). Cephalothorax Pb concentrations were consistently higher than tail-muscle concentrations, with the highest mean cephalothorax concentration observed in week 7 (2407.64 ± 142.35 µg/100 g). ART ANOVA (*n* = 5 shrimp per tissue per week) detected a significant effect of tissue (F = 29.12, *p* < 0.00001), whereas week (F ≈ 0.42, *p* = 0.887) and the week × tissue interaction (F ≈ 0.86, *p* = 0.610) were not significant.

Weekly mean values (±SE) for hemocytes, osmolality, NBT reduction, catalase activity, and lysozyme activity are summarized in Table 5. Hemocyte counts showed the highest mean value in week 8 (9.33 ± 0.67 × 10^6^ cells/mL), whereas osmolality was lowest in week 6 (1274.65 ± 35.73 mOsm/L). Catalase activity was highest in week 5 (2.313 ± 0.09 U/µL), and lysozyme activity was highest in week 6 (0.107 ± 0.02 U/µL). Kruskal–Wallis tests indicated temporal differences for hemocytes (H = 31.53, *p* < 0.0001), osmolality (H = 29.53, *p* = 0.0001), catalase activity (H = 20.48, *p* = 0.0046), and lysozyme activity (H = 14.67, *p* = 0.0405), but not for NBT reduction (H = 7.12, *p* = 0.4168). These temporal patterns are descriptive and cannot be attributed specifically to Pb under the single-system observational design.

Exploratory Spearman correlations based on weekly mean values did not remain statistically significant after Benjamini–Hochberg FDR correction. Raw associations included a negative correlation between osmolality and cephalothorax Pb concentration (*ρ* = −0.88, *p* = 0.0039, q = 0.077), a positive correlation between lysozyme activity and cephalothorax Pb concentration (ρ = 0.81, *p* = 0.0149, *q* = 0.149), and a negative one-week lagged correlation between catalase activity and outlet-water Pb concentration (*ρ* = −0.79, *p* = 0.036, q = 0.713). Other associations were weaker (*ρ* < 0.65). These unadjusted patterns are reported for exploratory context only and do not provide confirmatory evidence of Pb-specific physiological effects. The complete Spearman correlation matrix, including FDR-adjusted q-values, is provided in Table S3.

### 3.4. Root-Associated Bacterial Community Profiles in Aquaponic and Dune Reference Plants

Root-associated bacterial community profiles are described comparatively for plants sampled from the Pb-enriched aquaponic system and the dune reference site at week 8 (Figure 1). Across the six individual plant profiles, genera representing >1% relative abundance in at least one sample are displayed, whereas lower-abundance genera are grouped as “Other.” Because plants from the two groups were obtained from distinct environmental settings and were not part of a controlled experiment, the observed patterns are presented as descriptive environmental variation rather than Pb-specific effects.

The three aquaponic samples showed broadly similar community profiles, with *Neptunomonas*, *Altererythrobacter*, *Halomonas*, and taxa assigned to Halomonadaceae and Halobacteriaceae among the dominant groups. The dune reference samples displayed different dominance patterns, including greater relative abundances of *Sanguibacter* and *Halomonas*. As aquaponic and dune reference plants originated from distinct environmental contexts and were sampled at a single time point, no formal between-group statistical testing was conducted. Shannon diversity in individual aquaponic root samples ranged from 5.829 to 6.044 at the ASV level (Table S2).

### 3.5. Descriptive Comparison of Root-Associated Bacterial Communities

Root-associated bacterial communities were characterized at week 8 in the dune reference group (*n* = 3) and the Pb-enriched aquaponic group (*n* = 3). At the genus level, the aquaponic group showed 116 observed genera and a descriptive Shannon index of H′ = 4.24, whereas the dune reference group showed 106 observed genera and H′ = 4.00. These values provide a descriptive comparison only because samples were collected at a single time point and the two groups differed in environmental context.

Table 6 presents relative abundances of selected taxa that differ descriptively between groups. *Neptunomonas* was detected only in aquaponic root-associated samples (mean relative abundance: 10.87%), whereas *Halomonas* was more abundant in dune reference plants (7.13%) than in aquaponic plants (0.07%). Taxonomic assignments that were not consistent with bacterial 16S rRNA sequencing, including *Sanguinaria* and *Camelina sativa*, were excluded from biological interpretation and are identified in Table 6 for transparency.

## 4. Discussion

### 4.1. System-Level Pb Retention and Nutrient Dynamics

The aquaponic system showed substantial but temporally variable system-level Pb retention, with dissolved Pb concentrations averaging 75% lower at the hydroponic outlet than at the sampled inlet and reaching a maximum weekly inlet–outlet difference of 100% during week 6. This difference cannot be attributed solely to *S. portulacastrum*, because sedimentation, biofilter media, pipe surfaces, and biofilms may also adsorb, precipitate, or retain Pb. Pb accumulation in plant tissues indicates that *S. portulacastrum* participated in Pb retention; however, in the absence of a plant-free control system and a complete Pb mass balance, its specific quantitative contribution cannot be determined.

The observed temporal variation in system-level Pb retention is consistent with the influence of nutrient availability and water chemistry reported in other halophyte-based systems [22,23]. The negative exploratory correlations between Pb retention and inlet ammonium and total phosphorus may be compatible with changes in Pb bioavailability, adsorption, precipitation, or biological uptake. Previous studies have described competitive interactions involving ions and the formation of Pb–phosphate complexes under appropriate chemical conditions [24,25,26]. However, Pb speciation, precipitation processes, and component-specific uptake were not measured in the present study; therefore, these mechanisms cannot be identified from the observed correlations.

The positive exploratory association between Pb retention and outlet ammonium may also be related to biofilter- and hydroponic-loop processes that influence nitrogen dynamics in recirculating systems. Nevertheless, nitrification rates, ammonium transformation, and their direct effects on Pb retention were not quantified. Together, these patterns suggest that nutrient dynamics may be relevant to system-level Pb retention, but the limited number of weekly observations and the single-system observational design preclude causal inference. The exploratory multivariable model accounted for 55.5% of the observed variation in system-level Pb retention; given the limited number of weekly observations and the number of candidate predictors, this result should be interpreted as descriptive rather than predictive. Future replicated studies including Pb speciation, water-chemistry monitoring, and component-specific Pb mass balances are needed to assess whether management of nitrogen and phosphorus inputs can improve Pb retention performance.

### 4.2. Organ-Specific Pb Accumulation and Dynamic Allocation in Sesuvium portulacastrum

The variation in Pb concentrations among leaves, stems, and roots over time suggests differential distribution of Pb among plant organs in *S. portulacastrum*. The observed sequence is compatible with changes in tissue storage, translocation, dilution by growth, or differences in exposure among plant organs. Similar organ-specific patterns have been reported in halophytes exposed to metals, where tissue-level concentrations may reflect regulated sequestration, transport processes, and differences in tolerance among organs. However, because individual plants could contribute samples at more than one weekly time point and no direct measurements of Pb translocation or tissue biomass were performed, the temporal pattern cannot establish a defined internal allocation cycle. Further studies using individually tracked plants, biomass-normalized Pb burdens, and repeated measurements of translocation-related variables are needed to clarify organ-specific Pb handling under saline aquaponic conditions.

### 4.3. Temporal Variation in Antioxidant Variables of Sesuvium portulacastrum

Leaf antioxidant variables showed temporal variation during the monitoring period. Leaf POD and APX displayed changes across weeks that may be compatible with adjustment of antioxidant metabolism under changing conditions in the Pb-enriched aquaponic system. Similar antioxidant responses have been reported in halophytes exposed to metal-related stress. However, because the study lacked an uncontaminated aquaponic control and some plants may have contributed samples at more than one time point, the observed enzyme patterns cannot be attributed specifically to Pb or interpreted as discrete phases of detoxification [27,28].

CAT activity reached its highest mean value in week 8, coinciding with the highest foliar Pb concentration observed during the monitoring period. However, the absence of an uncontaminated aquaponic control, together with possible repeated sampling of individual plants, prevents interpretation of this temporal coincidence as Pb-specific antioxidant induction. AA also varied across weeks, whereas LYP did not show statistically significant temporal variation. These observations are consistent with temporal variability in antioxidant-related measurements under the conditions of the monitored system, but they do not establish the relative contribution of CAT, AA, or carotenoid-related processes to Pb tolerance [29,30,31].

Overall, the temporal patterns in Pb concentrations and antioxidant variables suggest that *S. portulacastrum* maintained biochemical activity under Pb-enriched aquaponic conditions. These observations provide exploratory evidence relevant to the selection of plant-based indicators for future replicated studies, but they do not establish Pb-specific antioxidant tolerance mechanisms.

### 4.4. Pb Accumulation and Physiological Responses in Litopenaeus vannamei

Pb accumulated preferentially in the cephalothorax, consistent with the presence of the hepatopancreas and other detoxification organs, as widely reported in metal-exposed shrimp [9,32]. This distribution remained stable over time, indicating that tissue-specific uptake pathways predominated over temporal variation in exposure concentrations.

Baseline values collected prior to exposure aligned with reported ranges for healthy *L. vannamei*, indicating that shrimp entered the system without detectable physiological impairment. Changes observed during weeks 1–8 therefore likely reflect subchronic temporal patterns associated with Pb-enriched conditions and other environmental factors within the aquaponic system.

Immune and physiological biomarkers showed distinct temporal patterns. Hemocyte counts increased sharply in weeks 6–8, while osmolality declined during the same period—a pattern directionally consistent with osmoregulatory disruption reported under metal stress in penaeid shrimp [33], though not statistically confirmed after FDR correction in the present study. Lysozyme (LZM) activity peaked in week 5 and showed a non-significant increasing tendency, consistent with patterns reported under chronic metal exposure. CAT exhibited a dual peak, in weeks 5 and 7, suggesting a temporal pattern of antioxidant activation, though this trend was not statistically significant after FDR correction. NBT remained stable, indicating that respiratory burst activity was less sensitive to Pb under these exposure conditions.

Although no correlations remained significant after FDR correction, the observed biological tendencies were directionally consistent: osmolality tended to decline as cephalothorax Pb increased, LZM activity showed a positive tendency with tissue Pb, and CAT displayed a suggestive, lagged relationship with waterborne Pb. While these patterns do not constitute statistical evidence of association, they are directionally coherent with responses reported under subchronic metal exposure in shrimp and warrant further investigation in replicated designs.

From a food-safety perspective, screening-level Pb concentrations detected in tail muscle of *L. vannamei* exceeded the 0.5 mg/kg wet-weight benchmark cited in EU Regulation 2023/915 and the Codex Alimentarius Standard for crustaceans at all weekly sampling points. Tail-muscle Pb concentrations ranged from 88.61 ± 5.12 to 356.54 ± 20.58 µg/100 g, equivalent to 0.89–3.57 mg/kg wet weight. However, because the 5-Carboxy-PADAP method was used as a screening approach and was not validated against certified reference materials or independently confirmed by ICP-OES, ICP-MS, or AAS, this regulatory comparison should be considered preliminary and requires confirmatory analysis before a definitive food-safety conclusion can be reached.

### 4.5. Root-Associated Microbial Restructuring Under Pb Exposure

Root-associated bacterial profiles from plants sampled in the Pb-enriched aquaponic system were dominated by *Neptunomonas*, *Altererythrobacter*, *Halomonas*, and taxa within Halomonadaceae and Halobacteriaceae—groups frequently reported in saline and metal-rich environments [34,35]. Some lineages within these broader taxa have been reported to tolerate metals or to possess capacities for biosorption, sulfide metabolism, or osmotic adjustment; however, the taxonomic profiles obtained here do not demonstrate these functions in the sampled root communities or their contribution to plant performance under combined salinity–metal stress [36].

In contrast, dune reference plants displayed higher relative abundances of taxa such as *Sanguibacter* and *Halomonas*, consistent with the concept that halophyte microbiomes reflect both environmental conditions and plant physiological status [37]. Although aquaponic and dune reference samples differed in environmental background and replication design, the observed differences in dominant genera are presented as descriptive patterns and cannot be attributed specifically to Pb exposure, aquaponic conditions, or any individual environmental factor.

At week 8, root-associated samples from the Pb-enriched aquaponic system contained 116 detected genera and had a Shannon diversity value of H′ = 4.24, whereas samples from the dune reference site contained 106 detected genera and had H′ = 4.00. These richness and diversity values are presented descriptively and should not be interpreted as evidence that Pb exposure altered overall microbial diversity. *Neptunomonas* was detected in aquaponic root samples (10.87%) and was not detected in dune reference samples. This marine genus has been reported to participate in contaminant transformation, and it occurred alongside *Ferrimonas*, *Navicula*, *Arcobacter*, and *Marinobacterium*—taxa previously associated with metal-impacted or saline environments. Conversely, *Halomonas* (7.13%) and *Cytophagia* (3.77%) were more abundant in dune reference samples and were rare or not detected in aquaponic root samples. Given the single time-point microbiome design, the distinct environmental backgrounds of the two sites, and the descriptive nature of the analysis, these compositional patterns should be considered exploratory observations requiring confirmation in replicated studies with temporal resolution.

In summary, the saline aquaponic system showed distinct patterns across plant, shrimp, and root-associated microbial measurements. Plants exhibited organ-specific Pb concentrations and temporal variation in leaf antioxidant variables. Shrimp showed tissue-specific Pb concentrations and variation in physiological variables across the sampling period. Root-associated bacterial profiles from aquaponic plants included taxa commonly reported from saline or metal-affected environments, while the aquaponic and dune reference samples displayed descriptive differences in community composition. Together, these multiscale observations illustrate the value of integrating plant, animal, and microbial indicators when characterizing Pb dynamics in saline aquaponic systems. They also identify halophyte–microbiome associations as a relevant topic for future controlled studies of phytoremediation-related processes and microbial functional roles under combined salinity and metal conditions.

## 5. Conclusions

This exploratory study shows that saline aquaponic systems supplied with naturally Pb-enriched water can be characterized through an integrated plant–animal–microbial monitoring framework. *S. portulacastrum* accumulated Pb in leaves, stems, and roots and may have contributed to Pb retention within the aquaponic system. However, because the study included only one aquaponic system and lacked a plant-free control, independent system-level replication, plant biomass measurements, and a complete Pb mass balance, the specific quantitative contribution of *S. portulacastrum* to system-level Pb retention could not be determined. Shrimp and root-associated microbial measurements provided complementary descriptive indicators of tissue Pb accumulation, physiological variation, and environmental community differences. Screening-level Pb concentrations detected in shrimp tail muscle exceeded the 0.5 mg/kg wet-weight benchmark cited in EU Regulation 2023/915 and the Codex Alimentarius across all weekly sampling points; however, confirmation using a validated atomic spectrometric method is required before definitive regulatory conclusions can be drawn. These findings underscore the need for routine Pb monitoring in aquaponic systems supplied by coastal groundwater sources and the value of incorporating multiscale bioindicators when managing heavy-metal risks in saline aquaponics. Future replicated studies should combine Pb speciation, water-chemistry monitoring, component-specific Pb mass balances, plant biomass measurements, and plant-free controls to clarify the mechanisms and relative contributions underlying Pb retention in saline aquaponic systems. These studies should also prospectively incorporate standardized shrimp growth-performance and survival endpoints, supported by individual- or cohort-level tracking, to assess the production implications of using naturally Pb-enriched water.

## Figures and Tables

**Figure 1:**
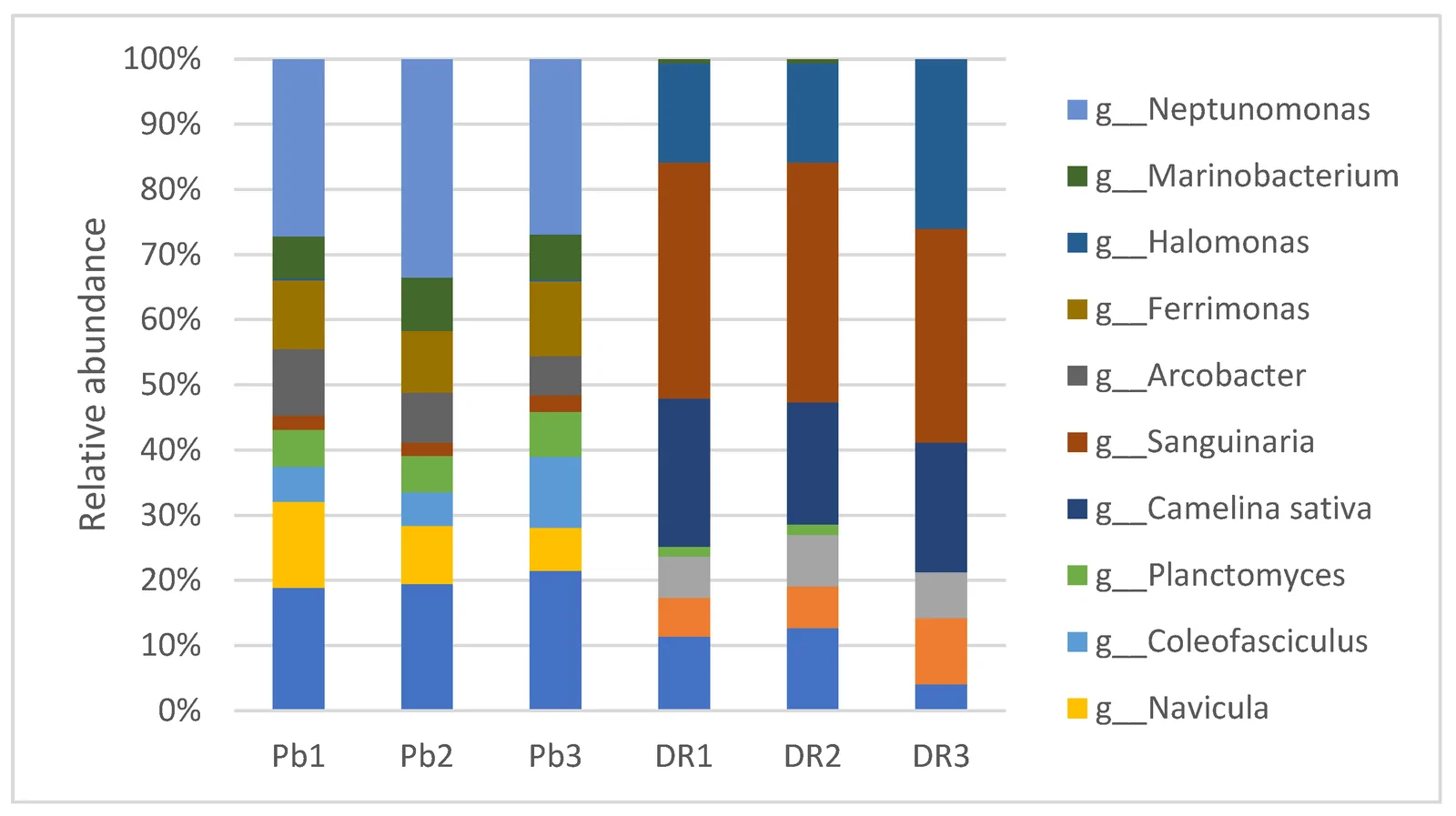
Relative abundance of bacterial genera representing >1% of total reads in at least one sample in root-associated samples of *Sesuvium portulacastrum* from the Pb-enriched aquaponic system (Pb1–Pb3) and the dune reference site (DR1–DR3), collected at week 8. Genera below the 1% threshold were grouped as “Other” for clarity. Each profile represents one individual plant. Dune reference plants (DR) are included for descriptive environmental comparison and do not constitute an experimental control group.

**Table 1:** Weekly dissolved Pb concentrations at the biofilter inlet and hydroponic outlet, and calculated system-level Pb retention in the saline aquaponic system.

Week	Inlet Pb (µg/L)	Outlet Pb (µg/L)	Pb-Retained (%)
1	4.00 ± 0.23	1.00 ± 0.06	75.0
2	6.00 ± 0.35	1.50 ± 0.09	75.0
3	5.50 ± 0.32	3.50 ± 0.20	36.4
4	8.50 ± 0.49	0.50 ± 0.03	94.1
5	3.00 ± 0.17	1.00 ± 0.06	66.7
6	2.50 ± 0.14	0.00 ± 0.00	100
7	13.00 ± 0.75	4.00 ± 0.23	69.2
8	8.00 ± 0.46	1.00 ± 0.06	87.5

**Table 2:** Tissue Pb concentrations (µg/100 g) in roots, stems, and leaves of *Sesuvium portulacastrum* collected from the saline aquaponic system.

Week	Leaf	Stem	Root
Baseline	20.72 ± 4.1	10.48 ± 1.7	18.57 ± 1.4
1	132.51 ± 12.8	109.97 ± 6.3	176.02 ± 10.2
2	94.32 ± 5.4	71.48 ± 4.1	143.00 ± 8.3
3	126.71 ± 17.3	98.49 ± 5.1	136.84 ± 10.9
4	160.92 ± 9.3	202.78 ± 11.7	65.13 ± 3.8
5	37.77 ± 2.2	243.34 ± 14.0	71.89 ± 4.2
6	48.05 ± 2.8	53.59 ± 3.1	82.95 ± 4.8
7	182.74 ± 10.6	132.34 ± 7.6	131.58 ± 9.6
8	197.88 ± 11.4	102.98 ± 5.9	136.84 ± 7.9

Baseline values correspond to plants collected from a natural coastal dune ecosystem with no known Pb contamination; these are environmental references, not experimental controls. *n* = 5 refers to five living plants sampled per week. Tissue portions were collected non-lethally, and plants remained in the system; consequently, some individuals may have contributed samples at more than one weekly time point. Samples represent within-system biological subsamples collected from the single aquaponic system and are not independent system-level replicates of Pb exposure.

**Table 3:** Antioxidant variables measured in leaf tissue of *Sesuvium portulacastrum* maintained in the Pb-enriched aquaponic system over eight weeks (mean ± SE, *n* = 5 within-system plant subsamples per week).

Week	Peroxidase (U/mg)	Ascorbate Peroxidase (U/mg)	Catalase (U/mg)	Ascorbic Acid (µg/mg)	Lycopene (mg/mg)
Baseline	66.01 ± 3.41	20.89 ± 2.24	191.15 ± 16.02	3.90 ± 0.38	17.65 ± 2.32
1	61.87 ± 1.08	16.49 ± 0.41	198.55 ± 6.67	4.53 ± 0.30	7.65 ± 0.56
2	61.53 ± 7.69	30.95 ± 1.75	213.05 ± 7.11	3.77 ± 0.11	11.81 ± 3.37
3	91.99 ± 8.36	17.24 ± 0.10	148.49 ± 30.17	3.20 ± 0.20	23.79 ± 5.94
4	28.64 ± 4.09	15.61 ± 0.27	154.46 ± 15.34	2.96 ± 0.28	15.29 ± 5.89
5	86.55 ± 6.66	21.84 ± 0.72	120.24 ± 32.85	5.14 ± 1.17	15.31 ± 3.02
6	55.60 ± 7.94	15.62 ± 2.99	98.28 ± 26.09	2.12 ± 2.03	17.78 ± 3.11
7	93.66 ± 5.51	30.22 ± 6.98	243.80 ± 20.76	4.96 ± 0.28	28.48 ± 9.49
8	48.21 ± 9.64	19.14 ± 0.86	313.51 ± 16.58	4.54 ± 0.34	19.32 ± 4.50

Baseline values correspond to leaf samples from *S. portulacastrum* collected from a natural coastal dune ecosystem with no known Pb contamination. These samples serve as environmental references and not as experimental controls. The *n* = 5 refers to five individual leaf subsamples collected per week within the single aquaponic system. Leaves were analyzed separately; leaf, stem, and root tissues were not pooled for antioxidant analyses. Because tissue sampling was non-lethal and some plants may have contributed leaf samples at more than one weekly time point, temporal comparisons are exploratory. Samples represent within-system biological subsamples collected from the single aquaponic system and are not independent system-level replicates of Pb exposure.

**Table 4:** Pb concentrations (µg/100 g) in tail muscle and cephalothorax of *Litopenaeus vannamei* before transfer (pre-exposure reference) and during eight weeks in the Pb-enriched aquaponic system (mean ± SE, *n* = 5 shrimp per tissue per week).

Week	Tail Muscle	Cephalothorax
Baseline	27.45 ± 6.99	46.44 ± 4.81
1	88.61 ± 5.12	721.12 ± 41.63
2	356.54 ± 20.58	785.75 ± 43.25
3	184.05 ± 10.63	667.36 ± 36.25
4	218.79 ± 12.56	998.32 ± 58.13
5	186.87 ± 10.98	1998.03 ± 115.24
6	133.44 ± 7.70	1996.58 ± 106.32
7	115.54 ± 6.67	2407.64 ± 142.35
8	263.87 ± 15.23	1993.69 ± 119.85

Baseline corresponds to Pb levels measured one week before shrimp entered the Pb-enriched aquaponic system. These values are provided solely as physiological reference and were not included in statistical analyses. *n* = 5 refers to five individual shrimp sampled once per week. Hemolymph was collected before euthanasia, and the same shrimp were subsequently used for Pb determination in tail muscle and cephalothorax. Analytical triplicates were averaged within each individual and were not treated as independent observations. The five-shrimp sampled per week represent within-system biological subsamples from the single aquaponic system and not independent system-level replicates of Pb exposure.

**Table 5:** Hemolymph physiological, oxidative, and immune biomarkers in *Litopenaeus vannamei* before transfer (pre-exposure reference) and during eight weeks in the Pb-enriched aquaponic system (mean ± SE, *n* = 5 shrimp per week).

Week	Hemocytes (cells/mL × 10^6^)	Osmolality (mOsm/L)	NBT Reduction Rate	Catalase (U/µL)	Lysozyme (U/µL)
Baseline	11.73 ± 1.10	1406.59 ± 41.19	1.48 ± 0.08	0.601 ± 0.08	0.080 ± 0.02
1	7.06 ± 0.61	1397.70 ± 49.47	1.03 ± 0.04	1.906 ± 0.16	0.028 ± 0.01
2	5.47 ± 1.18	1560.90 ± 31.95	0.82 ± 0.38	1.958 ± 0.16	0.044 ± 0.01
3	6.20 ± 0.6	1484.15 ± 23.56	0.95 ± 0.03	1.753 ± 0.10	0.038 ± 0.01
4	4.99 ± 0.87	1351.20 ± 32.78	0.95 ± 0.06	2.242 ± 0.15	0.050 ± 0.01
5	5.61 ± 1.20	1338.20 ± 49.09	0.91 ± 0.07	2.313 ± 0.09	0.066 ± 0.03
6	8.60 ± 0.66	1274.65 ± 35.73	1.04 ± 0.08	2.097 ± 0.17	0.107 ± 0.02
7	7.66 ± 1.55	1294.30 ± 11.04	0.91 ± 0.09	2.258 ± 0.17	0.091 ± 0.01
8	9.33 ± 0.67	1314.75 ± 39.96	0.85 ± 0.05	1.852 ± 0.19	0.051 ± 0.01

Baseline values correspond to hemolymph measurements collected one week before shrimp were transferred to the Pb-enriched aquaponic system. These values are provided solely as physiological reference and were not included in statistical analyses of weeks 1–8. *n* = 5 refers to five individual shrimp sampled once per week. Hemolymph was collected before euthanasia, and the same shrimp were subsequently used for Pb determination in tail muscle and cephalothorax. Analytical triplicates were averaged within each individual and were not treated as independent observations.

**Table 6:** Descriptive relative abundances (%) of selected taxonomic assignments in root-associated samples of *Sesuvium portulacastrum* from the dune reference site (DR1–DR3) and the Pb-enriched aquaponic system (Pb1–Pb3), collected at week 8. Shannon diversity index at genus level: dune reference H′ = 4.00; aquaponic H′ = 4.24.

Genus	DR1	DR2	DR3	DR Mean	Pb1	Pb2	Pb3	Aquaponic Mean
*Neptunomonas*	0.0	0.0	0.0	0.00	10.1	13.1	9.4	10.87
*Sanguinaria* ^†^	12.1	11.6	14.5	12.73	0.8	0.8	0.9	0.83
*Camelina sativa* ^‡^	7.6	5.9	8.8	7.43	0.0	0.0	0.0	0.00
*Halomonas*	5.1	4.8	11.5	7.13	0.1	0.0	0.1	0.07
*Navicula*	0.0	0.0	0.0	0.00	4.9	3.5	2.3	3.57
*Ferrimonas*	0.0	0.0	0.0	0.00	3.9	3.7	4.0	3.87
*Cytophagia*	3.6	3.8	3.9	3.77	0.0	0.0	0.0	0.00
*Arcobacter*	0.0	0.0	0.0	0.00	3.8	3.0	2.1	2.97
*Gemmatimonadetes*	5.5	5.3	8.8	6.53	0.3	0.3	0.4	0.33
*Marinobacterium*	0.2	0.2	0.0	0.13	2.4	3.2	2.4	2.67

^†^ *Sanguinaria* is a plant genus; this entry likely represents a taxonomic misassignment in the QIIME classification pipeline and should not be interpreted as a microbial taxon. ^‡^ *Camelina sativa* is a cultivated plant genus; this assignment does not represent a microbial taxon and is retained here for transparency.

## Data Availability

The data presented in this study are available on request from the corresponding author.

## Supplementary Materials

The following supporting information can be downloaded at: https://www.mdpi.com/article/10.3390/ani16193009/s1, Table S1: Weekly variation in water concentrations of ammonium, nitrite, nitrate, total phosphorus, and potassium at the inlet of the biofilter and outlet of the hydroponic unit in the aquaponic system; Table S2: Alpha diversity indices (Shannon and Simpson’s evenness) of root-associated bacterial communities in *Sesuvium portulacastrum* cultivated in Pb-enriched aquaponics (Pb-S) and in dune reference plants (DR). Table S3: Complete Spearman correlation matrix between physiological biomarkers of *Litopenaeus vannamei* and Pb concentrations in water and tissues.

## References

[1] Khedr A.I., Ghannam H.E. (2025). Evaluation of some heavy metals in water and health implications for fish consumers of the Great Cairo Sector of the Nile River. Sci. Rep..

[2] Martínez C.C.O., Juárez M.A., Vallejo-Peña J., López-López G., Ortíz-Arellano M.A., Zazueta-Ojeda I.H. (2024). Contenido de metales en músculo de camarones *Penaeus vannamei*, durante un ciclo de cultivo en Mazatlán, Sinaloa, México. Rev. Cienc. Mar UAS.

[3] World Health Organization (WHO) Intoxicación por Plomo. https://www.who.int/es/news-room/fact-sheets/detail/lead-poisoning-and-health.

[4] Sharma S.S., Dietz K.J., Mimura T. (2016). Vacuolar compartmentalization as indispensable component of heavy metal detoxification in plants. Plant Cell Environ..

[5] Alsherif E.A., Yaghoubi K.M., Crecchio C., Korany S.M., Sobrinho R.L., AbdElgawad H. (2023). Understanding the active mechanisms of plant (*Sesuvium portulacastrum* L.) against heavy metal toxicity. Plants.

[6] Zhou J., Zhang R., Wang P., Gao Y., Zhang J. (2024). Responses of soil and rhizosphere microbial communities to Cd-hyperaccumulating willows and Cd contamination. BMC Plant Biol..

[7] Imran I., Khan A.A., Kamal A., Alrefaei A.F., Zaman W. (2025). Utilization and characterization of microbes for heavy metal remediation. Pol. J. Environ. Stud..

[8] Wu Y.S., Huang S.L., Chung H.C., Nan F.H. (2017). Bioaccumulation of lead and non-specific immune responses in white shrimp (*Litopenaeus vannamei*) to Pb exposure. Fish Shellfish Immunol..

[9] Chen J., Xu W., Hu L., Luo Y., Bai Y., Wang Z. (2020). Growth and health status of Pacific white shrimp, *Litopenaeus vannamei*, exposed to chronic waterborne cobalt. Fish Shellfish Immunol..

[10] Zhang Y., Liu J., Zhuo H., Lin L., Li J., Fu S., Xue H., Wen H., Zhou X., Guo C. (2023). Differential toxicity responses between hepatopancreas and gills in *Litopenaeus vannamei* under chronic ammonia-N exposure. Animals.

[11] World Organisation for Animal Health (WOAH) (2021). Chapter 7.1: Introduction to Recommendations for Animal Welfare. Aquatic Animal Health Code.

[12] HACH Company (2014). Water Analysis Handbook.

[13] AOAC International (2000). Official Method 999.10. Lead, Cadmium, Zinc, Copper, and Iron in Foods by Graphite Furnace Atomic Absorption Spectrometry. Official Methods of Analysis of AOAC International.

[14] Song Y.-L., Hsieh Y.-T. (1994). Immunostimulation of tiger shrimp (*Penaeus monodon*) hemocytes for generation of microbicidal substances: Analysis of reactive oxygen species. Dev. Comp. Immunol..

[15] Chance B., Maehly A.C. (1955). Assay of catalases and peroxidases. Methods Enzymol..

[16] Aebi H. (1984). Catalase in vitro. Methods Enzymol..

[17] Nakano Y., Asada K. (1981). Hydrogen peroxide is scavenged by ascorbate-specific peroxidase in spinach chloroplasts. Plant Cell Physiol..

[18] Bunghez I.R., Raduly M., Doncea S., Aksahin I., Ion R.M. (2011). Lycopene determination in tomatoes by different spectral techniques (UV–Vis, FTIR and HPLC). Dig. J. Nanomater. Biostruct..

[19] Foyer C.H. (2017). Ascorbic acid. Antioxidants in Higher Plants.

[20] Lowry O.H., Rosebrough N.J., Farr A.L., Randall R.J. (1951). Protein measurement with the Folin phenol reagent. J. Biol. Chem..

[21] Wobbrock J.O., Findlater L., Gergle D., Higgins J.J. The aligned rank transform for nonparametric factorial analyses using only ANOVA procedures. Proceedings of the SIGCHI Conference on Human Factors in Computing Systems.

[22] Mani D., Kumar C. (2014). Biotechnological advances in bioremediation of heavy metals contaminated ecosystems: An overview with special reference to phytoremediation. Int. J. Environ. Sci. Technol..

[23] Sun Q., Zhou H., Xu C., Ba Y., Geng Z., She D. (2023). Effective adsorption of ammonium nitrogen by sulfonic-humic acid char and assessment of its recovery for application as nitrogen fertilizer. Sci. Total Environ..

[24] Cao X., Ma L.Q., Rhue D.R., Appel C.S. (2004). Mechanisms of lead, copper, and zinc retention by phosphate rock. Environ. Pollut..

[25] Anjum M.A.R., Iqbal S., Toba Z., Javaid S., Jamal A., Shafique M.A., Ullah M.S. (2023). Efficient lead sorption by ammonium phosphomolybdate: Experimental and density functional theory (DFT) studies. New J. Chem..

[26] Alam M.R., Islam R., Tran T.K.A., Le Van D., Rahman M.M., Griffin A.S., Yu R.M.K., MacFarlane G.R. (2021). Global patterns of accumulation and partitioning of metals in halophytic saltmarsh taxa: A phylogenetic comparative approach. J. Hazard. Mater..

[27] Navabpour S., Yamchi A., Bagherikia S., Kafi H. (2020). Lead-induced oxidative stress and role of antioxidant defense in wheat (*Triticum aestivum* L.). Physiol. Mol. Biol. Plants.

[28] Mehtari M., Talebi M., Sayed Tabatabaei B.E. (2025). The analysis of changes in antioxidant enzyme activity and gene expression caused by lead contamination in *Azolla caroliniana*. Int. J. Phytoremediat..

[29] Sainova G.A., Kaliyeva N.A., Yuldashbek D.K., Akbasova A.D. (2023). The influence of vermicompost and various concentrations of lead on the enzymatic activity of sierozem soils of Kazakhstan. Scientifica.

[30] Rudenko N.N., Vetoshkina D.V., Marenkova T.V., Borisova-Mubarakshina M.M. (2023). Antioxidants of non-enzymatic nature: Their function in higher plant cells and the ways of boosting their biosynthesis. Antioxidants.

[31] Pirasteh-Anosheh H., Samadi M., Kazemeini S.A., Ozturk M., Ludwiczak A., Piernik A. (2023). ROS homeostasis and antioxidants in the halophytic plants and seeds. Plants.

[32] Frías-Espericueta M.G., Páez-Osuna M.I., Osuna-Martínez O., Córdova-Leyva A., Romero-Geraldo J.A. (2016). Distribution of trace elements in tissues of shrimp species from the Gulf of California. Braz. J. Biol..

[33] Qiu J., Wang W.-N., Wang L.-J., Liu Y.-F., Wang A.-L. (2011). Oxidative stress, DNA damage and osmolality in the Pacific white shrimp, *Litopenaeus vannamei* exposed to acute low temperature stress. Comp. Biochem. Physiol. C Toxicol. Pharmacol..

[34] Suyamud B., Preechaporn W., Leelayuwat C., Wongyai S. (2017). Impact of heavy metal lead stress on polyamine levels in *Halomonas*. Sci. Rep..

[35] Wang R., Cui L., Li J., Li W. (2023). Factors driving the halophyte rhizosphere bacterial communities in coastal salt marshes. Front. Microbiol..

[36] Abdel-Razik M.A., Azmy A.F., Khairalla A.S., AbdelGhani S. (2020). Metal bioremediation potential of the halophilic bacterium, *Halomonas* sp. strain WQL9 isolated from Lake Qarun, Egypt. Egypt. J. Aquat. Res..

[37] Yin F., Li L., Song R., Wang Y., Qi H., Tan Y., Song N., Zhao T. (2021). Rhizosphere bacteria community and functions under typical halophyte species in arid China. PLoS ONE.

