# Supplementary material for: Lead Dynamics in Saline Aquaponics: Tissue Accumulation in Litopenaeus vannamei and Sesuvium portulacastrum and Root-Associated Bacterial Communities
Source: Animals (Basel). 2026 Sep 24;16(19):3009. doi: 10.3390/ani16193009 (PMC13663531; doi:10.3390/ani16193009)
Supplement: Supplementary file 1 [file animals-16-03009-s001.zip › animals-4571601-supplementary.pdf]

Table S1. Weekly variation in water concentrations of ammonium, nitrite, nitrate, total phosphorus, and potassium at the inlet of the biofilter and outlet of the hydroponic unit in the aquaponic system.

Water quality	Ammonium (NH_3)		Nitrite (NO_2^-)		Nitrate (NO_3^-)		Total Phosphorus (TP)		Potassium (K^+)	
Week	Inlet	Outlet	Inlet	Outlet	Inlet	Outlet	Inlet	Outlet	Inlet	Outlet
1	0.310	0.240	0.236	0.303	3.00	5.60	3.27	2.34	111.20	82.50
2	0.360	0.270	0.245	0.326	2.60	4.60	0.95	1.84	370.70	90.00
3	0.360	0.280	0.237	0.311	3.10	4.30	1.99	1.75	107.00	87.50
4	0.210	0.120	0.168	0.208	3.10	5.10	0.71	1.31	240.00	72.50
5	0.220	0.130	0.169	0.224	3.30	4.60	2.46	1.48	237.00	95.00
6	0.220	0.213	0.171	0.222	3.80	4.30	1.36	1.17	138.00	92.00
7	0.280	0.212	0.087	0.198	2.90	2.90	1.26	1.14	125.00	101.00
8	0.270	0.109	0.089	0.108	3.00	2.35	0.69	1.11	139.00	95.00

Values are expressed in mg/L. "Inlet" refers to the nutrient solution entering the integrated biofilter–hydroponic system each week, and "outlet" refers to the solution collected after circulation through the plants.

Table S2. Alpha diversity indices (Shannon and Simpson’s evenness) of root-associated bacterial communities in *Sesuvium portulacastrum* cultivated in Pb-enriched aquaponics (Pb-S) and in dune reference plants (DR).

Group	Shannon	Simpson_E
Pb-S	5.884	0.053
Pb-S	5.829	0.044
Pb-S	6.044	0.059
DR	6.192	0.021
DR	6.305	0.024
DR	3.975	0.260

Indices calculated at ASV level from Illumina MiSeq 16S rRNA sequencing. Genus-level Shannon diversity: Pb-S = 4.24; Dune reference plants (DR) = 4.00.

Table S3. Complete Spearman correlation matrix between physiological biomarkers of *Litopenaeus vannamei* and Pb concentrations in water and tissues.

**Response variable**	**Predictor variable**	**ϱ**	**p-value**	**q-value (FDR)**
Hemocytes	Pb-Inlet water	0.107	0.620	0.994
Hemocytes	Pb-Outlet water	-0.217	0.309	0.994
Hemocytes	Pb-Muscle	-0.116	0.590	0.994
Hemocytes	Pb-Cephalothorax	-0.422	0.040	0.454
Osmolality	Pb-Inlet water	0.066	0.759	0.994
Osmolality	Pb-Outlet water	-0.038	0.859	0.994
Osmolality	Pb-Muscle	-0.017	0.936	0.994
Osmolality	Pb-Cephalothorax	-0.880	0.004	0.077
NBT reduction	Pb-Inlet water	-0.177	0.409	0.994
NBT reduction	Pb-Outlet water	0.278	0.189	0.956
NBT reduction	Pb-Muscle	0.006	0.979	0.994

NBT reduction	Pb-Cephalothorax	-0.158	0.463	0.994
Catalase	Pb-Inlet water	0.159	0.458	0.994
Catalase	Pb-Outlet water	0.002	0.994	0.994
Catalase	Pb-Muscle	-0.142	0.509	0.994
Catalase	Pb-Cephalothorax	0.084	0.695	0.994
Catalase	Pb-Outlet water (lag-1)	-0.790	0.036	0.713
Lysozyme	Pb-Inlet water	0.101	0.639	0.994
Lysozyme	Pb-Outlet water	-0.032	0.881	0.994
Lysozyme	Pb-Muscle	0.276	0.191	0.956

q-values were calculated using the Benjamini-Hochberg false discovery rate (FDR) correction. No correlation remained significant after FDR correction (all $q > 0.05$). ρ = Spearman correlation coefficient; p = unadjusted p-value; q = FDR-adjusted p-value (Benjamini-Hochberg).
